# Whole genome sequencing for improved understanding of *Mycobacterium tuberculosis* transmission in a remote circumpolar region

**DOI:** 10.1017/S0950268819000670

**Published:** 2019-05-09

**Authors:** J. L. Guthrie, L. Strudwick, B. Roberts, M. Allen, J. McFadzen, D. Roth, D. Jorgensen, M. Rodrigues, P. Tang, B. Hanley, J. Johnston, V. J. Cook, J. L. Gardy

**Affiliations:** 1 School of Population and Public Health, University of British Columbia, Vancouver, Canada; 2 Yukon Communicable Disease Control, Health and Social Services, Government of Yukon, Whitehorse, Canada; 3 British Columbia Centre for Disease Control, Vancouver, Canada; 4 British Columbia Centre for Disease Control, Public Health Laboratory, Vancouver, Canada; 5 Department of Pathology, Sidra Medical and Research Center, Doha, Qatar; 6 Department of Health and Social Services, Government of Yukon, Whitehorse, Canada; 7 Department of Medicine, University of British Columbia, Vancouver, Canada

**Keywords:** Genomic epidemiology, remote setting, transmission networks, tuberculosis (TB)

## Abstract

Few studies have used genomic epidemiology to understand tuberculosis (TB) transmission in rural and remote settings – regions often unique in history, geography and demographics. To improve our understanding of TB transmission dynamics in Yukon Territory (YT), a circumpolar Canadian territory, we conducted a retrospective analysis in which we combined epidemiological data collected through routine contact investigations with clinical and laboratory results. *Mycobacterium tuberculosis* isolates from all culture-confirmed TB cases in YT (2005–2014) were genotyped using 24-locus Mycobacterial Interspersed Repetitive Units-Variable Number of Tandem Repeats (MIRU-VNTR) and compared to each other and to those from the neighbouring province of British Columbia (BC). Whole genome sequencing (WGS) of genotypically clustered isolates revealed three sustained transmission networks within YT, two of which also involved BC isolates. While each network had distinct characteristics, all had at least one individual acting as the probable source of three or more culture-positive cases. Overall, WGS revealed that TB transmission dynamics in YT are distinct from patterns of spread in other, more remote Northern Canadian regions, and that the combination of WGS and epidemiological data can provide actionable information to local public health teams.

## Introduction

Canada's tuberculosis (TB) rate has been decreasing overall, yet rates remain elevated in particular populations and regions. Recent outbreaks in two areas of Canada's North – Nunavik and Nunavut – resulted in annual incidence rates higher than many low-income countries [1, 2]. However, this is not the case in all circumpolar settings, where public health efforts have contributed to declining TB rates. From 2006 through 2012, Yukon Territory (YT) reported a rate of 12.1 cases per 100 000 population. While this is over twice the national average of 4.8 cases/100 000, it is the lowest rate amongst Canada's Northern territories (25.4/100 000 in the Northwest Territories, immediately east of YT, and 194.3/100 000 in Nunavut) [2, 3]. Alaska, located west of YT, has seen a sharp decrease in cases over the last few decades, reporting an average incidence of 8.1/100 000 (2006–2012), with most cases concentrated in rural communities – many inaccessible by road [2, 4]. Thus, while northern remote settings are often viewed similarly by population and public health programmes, it is clear that with respect to TB, there are significant differences across these regions, likely explained by a combination of the robustness of regional public health, access to appropriate housing, geography, intra-community movement and the populations themselves [5]. Understanding the unique epidemiology of TB in each region is therefore vital to delivering tailored interventions to drive rates in circumpolar settings closer to the World Health Organization's elimination goals.

Genotyping programmes have provided significant insights into the molecular epidemiology of TB in many low-incidence countries, helping to detect outbreaks [6, 7], and more recently, genome sequencing has dramatically improved our understanding of both clustering and TB transmission in communities worldwide [8–11]. However, only two studies to date have used this genomic epidemiology approach to examine transmission in remote Northern locations: one in Nunavik, Québec [12] – an Arctic region of Canada's North, and a second in Greenland, which used genomics to detect ‘hotspot cases’ responsible for chains of transmission [13]. To better understand the patterns of TB transmission in YT, we sequenced *Mycobacterium tuberculosis* (*Mtb*) genomes from all culture-positive TB diagnoses in YT over a 10-year period – the first genomic epidemiology study of TB in this region. Recognising that in contrast to many other northern regions in Canada, year-round highway access and multiple airports facilitate travel between YT and its southern neighbour, British Columbia (BC), we also examined YT *Mtb* genomes in the context of *Mtb* genomes sequenced in BC during the same time period. This unique cross-border comparison is possible as the BC Centre for Disease Control (BCCDC) and the BC Public Health Laboratory (BCPHL) are contracted by YT to provide TB services such as laboratory diagnostics and case management support, and both jurisdictions access a shared data repository, thus allowing us to identify the chains of transmission within and across YT/BC borders, and to fully describe the genomic epidemiology of TB in this remote circumpolar region.

## Methods

### Study setting and design

**Fig. 1.**
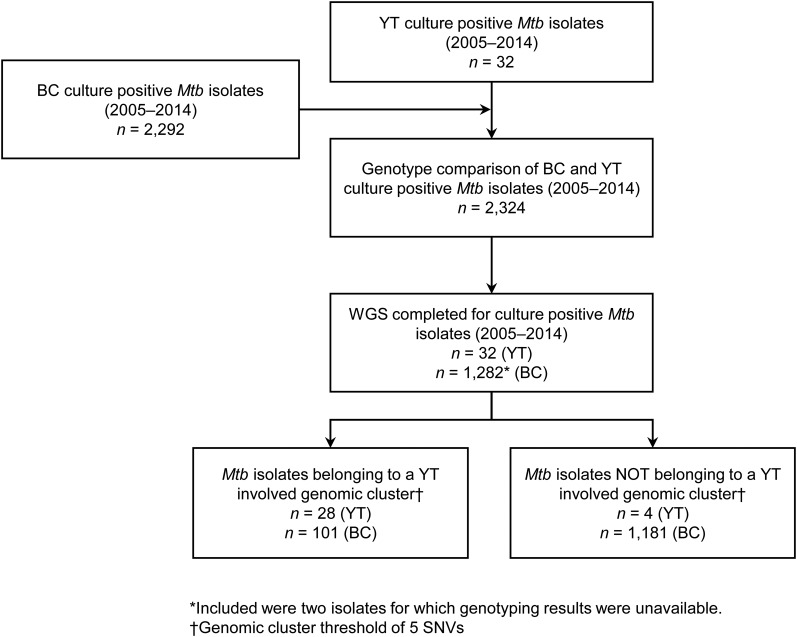
Study sample flow diagram summarising the number of isolates from British Columbia (BC) and Yukon Territory (YT) belonging to a YT involved genomic cluster based on a five single-nucleotide variants (SNVs) threshold.

YT is the sparsely populated (0.1 persons/km^2^) [14], most Northwestern region of Canada, immediately north of BC. All TB cases diagnosed in YT are reported to the Yukon Communicable Disease Control (YCDC), and those in BC to the BCCDC. Care and treatment of individuals diagnosed with TB is the responsibility of YCDC, in partnership with Yukon Government Community Nursing and includes contact investigations (CIs) for newly diagnosed cases. The BCPHL receives all *Mtb* isolates for both YT and BC, and conducts routine diagnostic testing, universal 24-locus Mycobacterial Interspersed Repetitive Units-Variable Number of Tandem Repeats (MIRU-VNTR) genotyping, and whole genome sequencing (WGS) on request. The study population (Fig. 1) included all YT culture-positive TB cases from 2005 through 2014 (*n* = 32), which were compared with TB cases diagnosed in BC during the same time period (*n* = 2292), for which the BC study population has been previously described [15]*.*

Ethics approval for this study was granted by the University of British Columbia (certificate #H12-00910).

### Case-level information

Case-level clinical and demographic data, as well as epidemiological data collected during routine CIs, for all TB cases from BC and YT were extracted from the integrated Public Health Information System (iPHIS). To classify community type for BC cases into metro (>190 000), urban/rural (40 001–190 000), rural (10 001–40 000) and remote (⩽10 000) groups, we used the population density of the geographic service area in which each case resided. YT community types were classified by home postal code, with the second digit ‘1’ in the forward sortation area indicating urban/rural, and a ‘0’ indicating a remote community.

### Laboratory methods

All *Mtb* isolates were obtained from specimens submitted to the BCPHL for routine diagnostic and phenotypic susceptibility testing. Isolates were revived from archived frozen stocks, DNA was extracted and 24-locus MIRU-VNTR genotyping was performed as previously described [15]. Isolates lacking an amplicon peak at any locus were repeated with newly extracted DNA, and where there remained no peak at a single locus, the locus was coded as missing data and included in the analyses. All 32 culture-positive isolates of 38 notified cases in YT during the study period were successfully genotyped. These results were compared to genotypes of all culture-positive *Mtb* isolates from BC over the same period [15]. WGS was completed for all 32 YT isolates as well as 1284 BC isolates – which included all isolates genotypically clustered by MIRU-VNTR to a YT isolate. WGS was completed using 125 bp paired-end reads on the Illumina HiSeqX platform at Canada's Michael Smith Genome Sciences Centre (Vancouver, BC).

### WGS analysis

The bioinformatics pipeline developed by Oxford University and Public Health England was used to analyse the resulting fastq files [16]. Reads were aligned to the *Mtb* H37Rv reference genome (GenBank ID: NC000962.2), with an average of 92% of the reference genome covered. Single-nucleotide variants (SNVs) were identified across all mapped non-repetitive sites. Genomic clusters were defined independently of MIRU-VNTR clusters and a unique identifier (WClustID) was assigned where isolates differed by ⩽5 SNVs – a threshold reflecting recent local transmission [9]. Concatenated SNVs combined with epidemiological data collected through routine CIs and consultation with YCDC public health authorities were used to generate temporal transmission networks. Major lineage was predicted for each sequenced isolate based on lineage-defining SNVs [17], and *in silico* antibiotic resistance was predicted as previously described [18]. Fastq files for all genomes are available at NCBI under BioProject PRJNA413593 and PRJNA49659.

### Statistical analysis

We calculated descriptive statistics for basic demographic and clinical information across two categories: (i) all cases diagnosed within YT, and (ii) cases diagnosed in BC residents within five SNVs of a YT case and classified as ‘Related’ (BC^R^). Univariable analysis used the *t*-test for comparisons of mean age, and categorical variables were compared using *χ*^2^ or Fisher's exact test where appropriate. The frequency for which a MIRU-VNTR pattern was observed within the YT and/or BC^R^ populations was described, and to place MIRU-VNTR genotypes in the wider context of BC as a whole, we also compared genotypes to BC isolates not closely related to YT isolates based on genomic distance thresholds (>5 SNVs) and classified these as ‘Not Related’ (BC^NR^). A dendrogram based on 24-locus MIRU-VNTR genotyping patterns was generated using the categorical (Hamming) distance and UPGMA (unweighted pair group method with arithmetic mean) algorithm. All statistical analyses were done in R v3.4.1.

## Results

### MIRU-VNTR and WGS provide different estimates of clustering

**Fig. 2.**
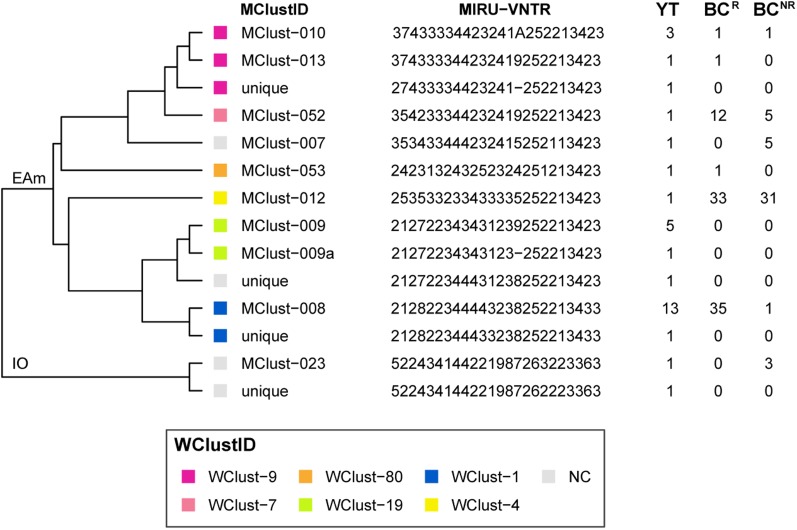
Dendrogram based on 24-locus MIRU-VNTR genotypes of *Mycobacterium tuberculosis* isolates collected in Yukon Territory (YT) from 2005 through 2014. MIRU-VNTR clusters (⩾2 isolates) were assigned a unique MClustID. The number of times each MIRU-VNTR pattern was observed in the YT, BC^R^ (within 0–5 SNVs of a YT isolate) and BC^NR^ (>5 SNVs to a YT isolate) populations are indicated in the right-hand side columns. Coloured squares represent the whole genome sequencing cluster, and isolates >5 SNVs from isolates in YT or British Columbia (BC) were considered not genomically clustered (NC). WGS clusters (⩾2 isolates within 0–5 SNVs) were assigned a unique WClustID independent of MIRU-VNTR. Lineage indicated at root. Abbreviations: EAm, Euro-American; IO, Indo-Oceanic. Order of loci: MIRU 04, MIRU 26, MIRU 40, MIRU 10, MIRU 16, MIRU 31, 424, 577, 2165, 2401, 3690, 4156, 2163, 1955, 4052, MIRU 02, MIRU 23, MIRU 39, MIRU 20, MIRU 24, MIRU 27, 2347, 2461, 3171.

From 2005 through 2014, 32 individuals were diagnosed with culture-positive TB in YT. MIRU-VNTR genotyping grouped 21 of these cases into three clusters (3–13 YT isolates/cluster), yielding a clustered proportion of 65.6% within the territory. One YT isolate had an untypable locus yet matched a cluster unique to YT for the other 23 typable loci. Six YT isolates had MIRU-VNTR patterns that were unique amongst the YT population yet clustered with isolates in BC, bringing the total number of MIRU-VNTR clusters across both jurisdictions containing at least one YT case to nine (Fig. 2). Four YT isolates remained unclustered after comparison with all BC isolates. All four were within one or two loci of a YT and/or BC genotype cluster.

**Fig. 3.**
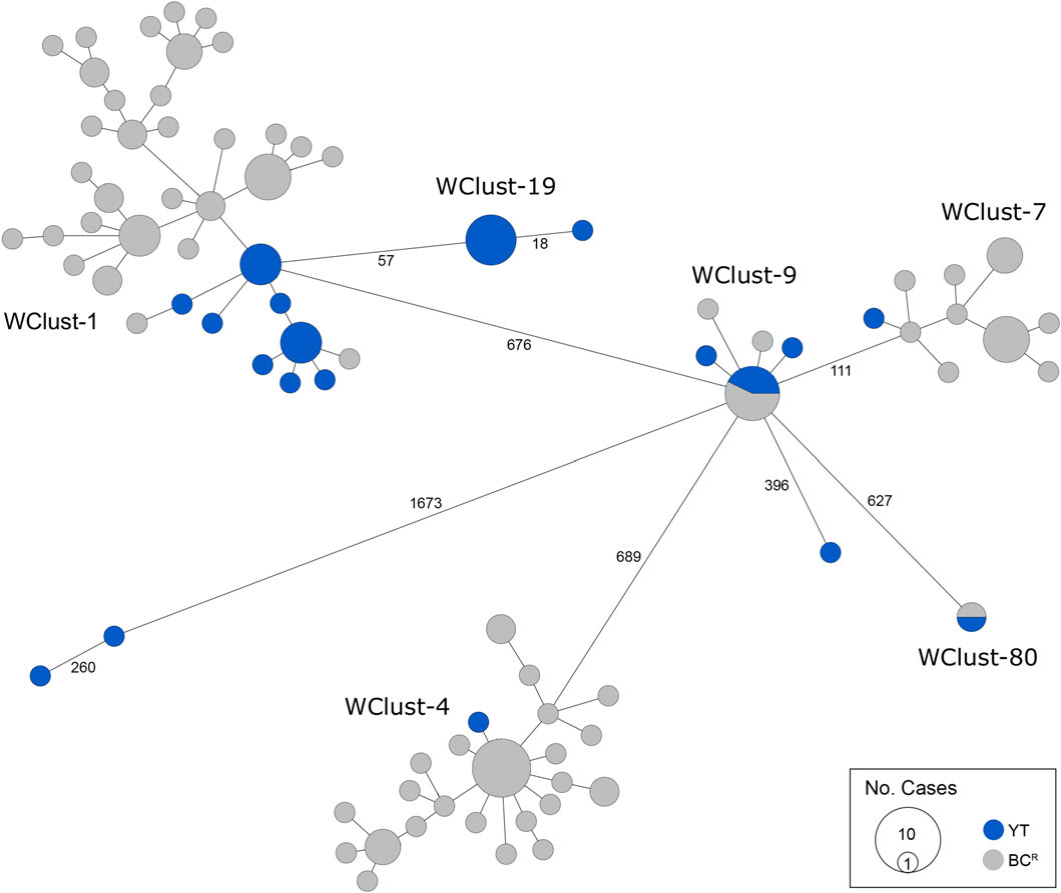
Minimum-spanning tree based on whole genome sequences of *Mycobacterium tuberculosis* (*Mtb*) isolates from the Yukon Territory (YT), Canada study population (*n* = 32) and closely related (five single-nucleotide variants (SNVs) threshold) isolates from British Columbia (BC) (*n* = 101). The size of each circle is proportional to the number of isolates, and circles are coloured in blue to represent the YT study population and grey for the BC population. Unique cluster identifiers (WClustID) are indicated for isolates in genomic clusters. The number of SNVs between isolates with >5 SNVs are indicated along the connecting branches.

Genomics provided a higher resolution view of clusters suggestive of recent transmission, merging several MIRU-VNTR clusters that differed by a single locus or had an untypable locus into single groups supported by CI data, and in other cases revealing that MIRU-VNTR clustered isolates, such as those belonging to MClust-023, were not truly clustered in a way that would suggest recent local transmission (Fig. 2). Using a five SNV threshold, we identified six genomic clusters with at least one YT case, involving a total of 28 YT and 101 BC^R^ isolates and ranging from two to 59 isolates (Fig. 3). Another YT isolate was within 20 SNVs of a genomic cluster, while the remaining three isolates were >200 SNVs away from any other YT isolate. By WGS, the clustered proportion was 28/32 (87.5%) when YT isolates were considered alongside BC isolates, and 25/32 (78.1%) considering only isolates among YT residents. With the exception of two Indo-Oceanic lineage isolates, all other YT isolates (94.1%) belonged to the Euro-American lineage. One of the Indo-Oceanic lineage isolates was phenotypically resistant to isoniazid (0.4 µg/ml) due to a *katG* S315T mutation, while the remaining isolates were susceptible to all first-line antibiotics.

### Genomically related cases across jurisdictions are similar clinically

Comparing all YT cases to the genomically related BC^R^ cases (*n* = 101), we found similar characteristics across both populations, including the mean age of 45.8 years (standard deviation (s.d.) ± 16.7) and 46.8 years (s.d. ± 11.9) for YT and BC^R^ individuals, respectively. Both groups were predominantly Canadian-born, with 93.8% of the YT study population and 88.9% of BC^R^ persons born in Canada (Table 1). The proportion of individuals with a clinical presentation associated with TB transmission was high in the YT and BC^R^ populations, with respiratory TB diagnosed in 90.6% of YT and 89.1% of BC^R^ individuals. Likewise, the smear-positive TB proportion was high – >82% in YT and BC^R^ persons. Of note, the proportion of individuals with cavitary TB was over 1.5× higher in the YT population compared with BC^R^ individuals, with cavitary disease in 37.5% (12/29) of YT persons (*P* = 0.099). With respect to risk factors for transmission [19], the majority of individuals (YT: 71.9%, BC^R^: 61.5%) reported ⩾1 risk factor (HIV, illicit drug use or alcohol misuse). Reflecting the differing demographics between the two settings, the majority of YT individuals resided in remote (84.4%) regions, compared with those in BC^R^ where the majority resided in metro areas (82.2%).

**Table 1.** Demographic and clinical characteristics of culture-positive cases across Yukon and genomically related cases in British Columbia, Canada, 2005–2014^a^

	No. cases (%)		
Characteristic	YT	BC^R^	*P*-value
Totals	*n* = 32	*n* = 101	
Age, years			
0–24	3 (9.4)	1 (1.0)	0.086
25–44	10 (31.2)	40 (39.6)	
45–64	14 (43.8)	50 (49.5)	
65+	5 (15.6)	10 (9.9)	
Gender			
Male	21 (65.6)	69 (68.3)	0.777
Community			
Metro	0 (0.0)	83 (82.2)	<0.001
Urban/rural	5 (15.6)	7 (6.9)	
Rural	0 (0.0)	8 (7.9)	
Remote	27 (84.4)	3 (3.0)	
Birthplace^b^			
Canada	30 (93.8)	88 (88.9)	0.734
Disease site			
Respiratory	29 (90.6)	82 (81.2)	0.344
Non-respiratory	3 (9.4)	11 (10.9)	
Respiratory + non-respiratory	0 (0.0)	8 (7.9)	
Respiratory^c^ smear			
Positive	23 (82.1)	76 (83.5)	1.000
Cavitary disease			
Yes	12 (37.5)	23 (22.8)	0.099
Risk factors^d^			
None	9 (28.1)	30 (38.0)	0.325
⩾1	23 (71.9)	49 (62.0)	

YT, Yukon Territory; BC^R^, British Columbia Related (Mtb isolates ⩽5 SNVs to study population); SNVs, single-nucleotide variants.

a Percentages have been rounded and may not total to 100%.

b Data unavailable *n* = 2 (BC^R^).

c Excluded ‘other respiratory’ sites, e.g. pleura.

d Risk factors = HIV, illicit drug use or alcohol misuse; data unavailable for one or more risk factors in BC^R^ population (*n* = 22).

### Transmission reconstruction

**Fig. 4.**
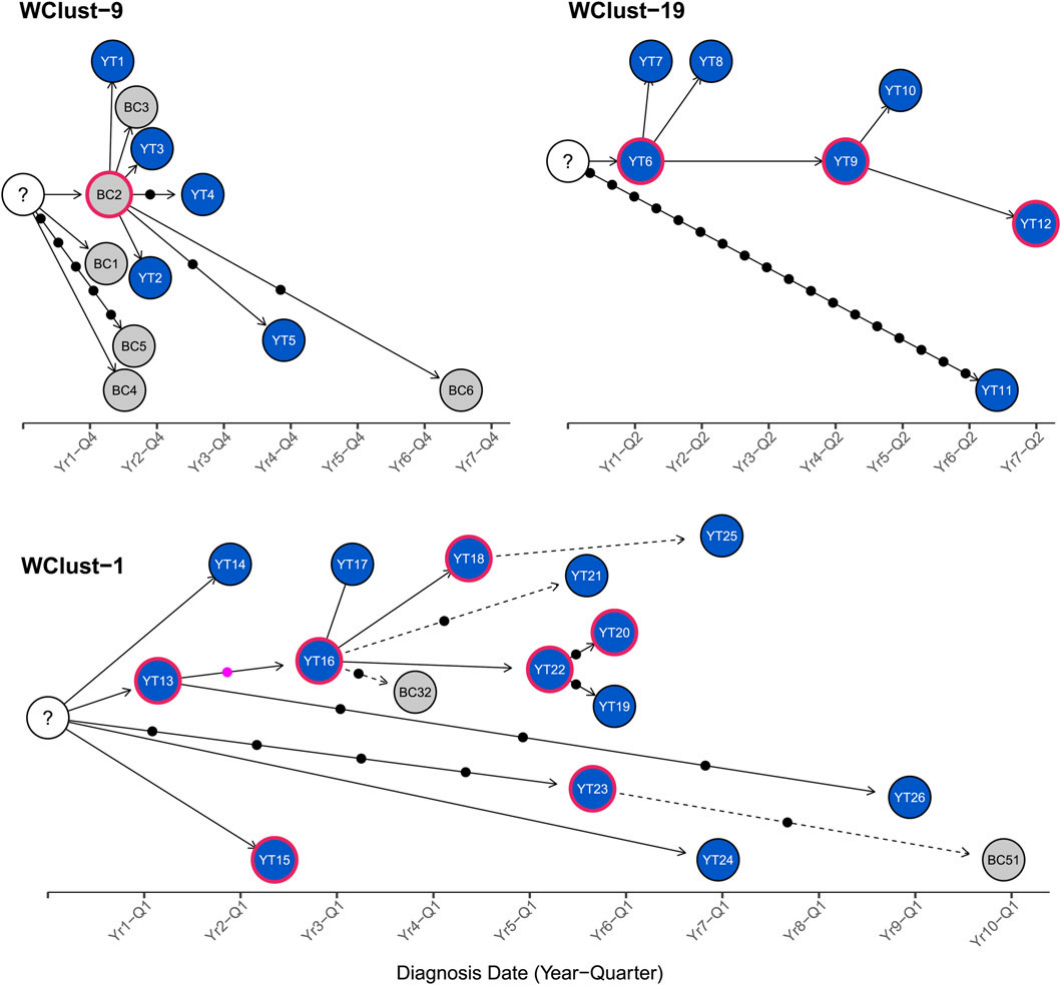
Transmission networks of three *Mycobacterium tuberculosis* genomic clusters (based on five single-nucleotide variants (SNVs) threshold) representing transmission, Yukon Territory (YT), Canada (2005–2014). Blue circles represent YT isolates and grey British Columbia (BC) isolates. A red outline around a circle represents acid-fast bacillus smear-positive + cavitary disease. Solid lines indicate strong epidemiological linkages, and dashed lines indicate weak epidemiological linkages. SNVs acquired over time are represented by dots between isolates. The pink dot represents the presence of a minority-variant which is passed on to all isolates in the subsequent transmission chain.

To characterise person-to-person spread of TB within YT, we constructed temporal transmission networks using WGS results combined with epidemiological data for the three genomic clusters with transmission between or to numerous YT persons – WClust-1, WClust-9 and WClust-19 (Fig. 4). Although *Mtb* isolate YT13 is above the five SNV threshold set for recent transmission, it is within 18 SNVs of WClust-19 – a cluster genotypically and genomically unique to the YT population – and was therefore included in the reconstruction figure, recognising this case likely represents reactivation of a previously acquired infection with a strain circulating within YT. For WClust-1, a large cluster with discrete minimum spanning tree branches in both YT and BC, we included only the branch of YT isolates, together with the two closely related BC isolates (Fig. 3).

Each of the three clusters differs slightly. WClust-19 is the only cluster exclusively comprising YT individuals, whereas WClust-1 and WClust-9 had one or more BC persons with related isolates. Within WClust-1 the BC cases may have acquired TB from a YT individual, whereas in WClust-9 a BC individual likely transmitted TB to a number of BC and YT cases. SNV distances ranged within clusters; however, WClust-19 saw no genomic variation in the transmission chain stemming from YT8, despite up to 6 years between disease acquisition and diagnosis. WClust-9 has four BC isolates 0–5 SNVs from those in YT (Fig. 4). However, with the exception of BC2, there are no known epidemiological connections between these cases that would suggest a common source not identified through CIs.

WClust-1 represents the largest YT cluster. CIs revealed that many of the individuals were social contacts of one another, with at least two individuals suspected of giving rise to multiple secondary cases. Here, genomics identified a minority variant (at the SNV site, 15% of reads had adenine (A) and 85% were cytosine (C)) in YT18, whereas in the subsequent cases, the SNV was fully fixed, confirming this individual as the most likely source for the cases that followed (online Supplementary Fig. S1). Genomic data also confirmed the inclusion of three Yukon (YT23, YT25 and YT27) and two BC isolates (BC31 and BC49) in this cluster, despite no apparent epidemiological linkages to each other or other cluster members.

While each of the three genomic clusters had unique features, all had at least one individual source of multiple culture-positive secondary cases, and all spanned several years, with some individuals progressing rapidly to active disease, and others reactivating after a long period of latency.

## Discussion

We describe the genomic epidemiology of TB in Northwestern Canada over a 10-year period, finding that persons diagnosed with TB were largely Canadian-born with Euro-American lineage isolates, with nearly all cases attributable to transmission within Canada, consistent with the epidemiology of TB elsewhere in Canada's North [1, 12].

Genomic data, combined with detailed epidemiological data, allowed us to reconstruct likely transmission pathways among the three large clusters. We found that, as is true for a number of infectious diseases, a small number of individuals account for a disproportionate number of secondary cases – the phenomenon of ‘super-spreaders’ [20]. Understanding the risk factors and epidemiological characteristics driving super-spreading in a community is important for better prioritising TB prevention and care programmes. In our YT study population, the proportion of individuals with clinical risk factors frequently associated with transmission, such as cavitary disease and smear positivity [19], was quite high, and anecdotal evidence from the local public health team suggested that delays in diagnosis might have also contributed to transmission. A recent publication [21] discussed the various drivers of TB transmission outside clinical risk factors, including diagnostic delays, which increase the potential for disease progression and transmission [22, 23], particularly amongst highly mobile, socially connected and infectious individuals.

Given the shared border between YT and BC, we also examined transmission across jurisdictions. Including genomically related BC isolates increased our estimate of clustering for YT isolates, suggesting that estimates derived from individual provincial or territory data alone likely underestimate transmission rates in relation to remote settings. Cross-border transmission appears to occur in both directions – in several cases YT residents likely transmitted to BC residents via social/community connections with YT residents reporting travel/residential histories in both Northern BC communities and larger metropolitan regions. Additionally, three YT cases had isolates that clustered only with BC isolates and likely acquired their infections within BC, while a BC source was linked to six YT cases in WClust-9. Previous studies [12, 13] of TB transmission in circumpolar settings saw genomically clustered isolates localised to specific communities; here, we observe the opposite, with transmission occurring across geographic boundaries. This underscores the notion that not all circumpolar TB transmission is the same, and while community-level interventions may be appropriate for some settings, investigating TB transmission in settings like YT requires intra-jurisdictional cooperation and multi-sectorial interventions.

Given the low genomic variation between cases, with most cases differing by 0–1 SNVs, our cluster reconstructions were only possible thanks to the detailed epidemiological information collected by the local public health team. Such minimal variation across multiple hosts over many years is not uncommon, and has been previously described in outbreaks elsewhere in Canada [11]. Our observation reinforces the need for comprehensive CI data coupled to genomics to fully understand regional epidemiology, though it is important to note that because genomic studies currently require *Mtb* culture, culture-negative TB cases are excluded from reconstructions. These cases are less likely to contribute to transmission due to low bacterial loads but cannot be completely excluded. TB diagnoses prior to the study period are also not captured here.

Understanding TB transmission dynamics is a key to the design and delivery of effective evidence-based interventions to prevent the continuing spread of TB, and WGS will be an integral part of future investigations into the unique patterns of TB spread in a given region. It offers more focused epidemiological information than traditional laboratory methods, such as MIRU-VNTR, with a faster turn-around-time and at roughly the same costs, and enables *in silico* resistance prediction [24]. It also permits distinguishing between reactivation of a historically acquired latent TB infection and TB resulting from recent transmission [25]. This is particularly important in small populations with isolates sharing high degrees of genotypic relatedness, such as YTs, where CI alone may not be able to differentiate these two scenarios. Nevertheless, disparities in basic laboratory services, turn-around-times and access to new technologies often exist between circumpolar territories and the rest of the country and it is likely to be some time before WGS moves out of the specialised reference laboratory landscape and into real-time use in remote settings. In the interim, a commitment by reference laboratories supporting these regions is needed to ensure that *Mtb* isolates from remote territories are included in WGS efforts, which will help bridge the gap and provide the same opportunity as the rest of the country to impact public health management of TB cases.

While the immediate impact of WGS on contact-tracing practices and annual TB incidence rates is yet to be seen, as we collect more data and build more transmission networks for a given region, we can begin to understand the fine-scale trends driving transmission, and deploy interventions targeted to meet the region's specific needs. These may include: (i) targeted messaging to clinicians in remote settings to ‘think TB’, many of whom may not have seen a case of TB; (ii) enhanced CIs around individuals whose presentation and molecular epidemiology is consistent with that of a super-spreader; and (iii) implementing a framework to facilitate working across regional or provincial jurisdictions to jointly manage outbreaks – supported through the use of genomics. We therefore recommend routine WGS of TB cases from circumpolar regions to better understand the unique regional dynamics driving transmission and assess ongoing levels of transmission in these settings.

## Supplementary material

For supplementary material accompanying this paper visit https://doi.org/10.1017/S0950268819000670. click here to view supplementary material

## References

[1] Public Health Agency of Canada and Canadian Lung Association/Canadian Thoracic Society (2014) *Canadian Tuberculosis Standards – 7th edition* Available at https://www.canada.ca/en/public-health/services/infectious-diseases/canadian-tuberculosis-standards-7th-edition/edition-22.html (Accessed 1 October 2018).

[2] Bourgeois A-C (2018) Tuberculosis in the circumpolar region, 2006–2012. The International Journal of Tuberculosis and Lung Disease 22, 641–648. 29862948 10.5588/ijtld.17.0525

[3] Vachon J, Gallant V and Siu W (2018) *Tuberculosis in Canada*, 2016. Public Health Agency of Canada. Report No.: Volume 44–3/4. 10.14745/ccdr.v44i34a01 PMC6449093 31007614

[4] State of Alaska (2017) *Tuberculosis in Alaska*, 2016 *Annual Report*.

[5] Dehghani K (2018) Determinants of tuberculosis trends in six Indigenous populations of the USA, Canada, and Greenland from 1960 to 2014: a population-based study. The Lancet Public Health 3, e133–e142. 29426597 10.1016/S2468-2667(18)30002-1

[6] Anderson LF (2014) Transmission of multidrug-resistant tuberculosis in the UK: a cross-sectional molecular and epidemiological study of clustering and contact tracing. The Lancet Infectious Diseases 14, 406–415. 24602842 10.1016/S1473-3099(14)70022-2

[7] Lalor MK (2017) Recent household transmission of tuberculosis in England, 2010–2012: retrospective national cohort study combining epidemiological and molecular strain typing data. BMC Medicine 15, 105. 28606177 10.1186/s12916-017-0864-y PMC5469076

[8] Gardy JL (2011) Whole-genome sequencing and social-network analysis of a tuberculosis outbreak. The New England Journal of Medicine 364, 730–739. 21345102 10.1056/NEJMoa1003176

[9] Walker TM (2013) Whole-genome sequencing to delineate *Mycobacterium tuberculosis* outbreaks: a retrospective observational study. The Lancet. Infectious Diseases 13, 137–146. 23158499 10.1016/S1473-3099(12)70277-3 PMC3556524

[10] Roetzer A (2013) Whole genome sequencing versus traditional genotyping for investigation of a *Mycobacterium tuberculosis* outbreak: a longitudinal molecular epidemiological study. PLoS Medicine 10, e1001387. 23424287 10.1371/journal.pmed.1001387 PMC3570532

[11] Mehaffy C (2014) Marked microevolution of a unique *Mycobacterium tuberculosis* strain in 17 years of ongoing transmission in a high risk population. PLoS ONE 9, e112928. 25405861 10.1371/journal.pone.0112928 PMC4236100

[12] Lee RS (2015) Reemergence and amplification of tuberculosis in the Canadian Arctic. Journal of Infectious Diseases 211, 1905–1914. 25576599 10.1093/infdis/jiv011

[13] Bjorn-Mortensen K (2016) Tracing *Mycobacterium tuberculosis* transmission by whole genome sequencing in a high incidence setting: a retrospective population-based study in East Greenland. Scientific Reports 6, 33180. 27615360 10.1038/srep33180 PMC5018808

[14] Government of Canada SC (2012). *Statistics Canada: 2011 Census Profile* Available at http://www12.statcan.gc.ca/census-recensement/2011/dp-pd/prof/details/Page.cfm?Lang=E&Geo1=PR&Code1=60&Geo2=PR&Code2=01&Data=Count&SearchText=Yukon&SearchType=Begins&SearchPR=01&B1=All&GeoLevel=PR&GeoCode=60 (Accessed 3 October 2018).

[15] Guthrie JL (2017) Molecular epidemiology of tuberculosis in British Columbia, Canada: a 10-year retrospective study. Clinical Infectious Diseases 66, 849–856. 10.1093/cid/cix906 PMC5850024 29069284

[16] Walker TM (2015) Whole-genome sequencing for prediction of *Mycobacterium tuberculosis* drug susceptibility and resistance: a retrospective cohort study. The Lancet. Infectious Diseases 15, 1193–1202. 26116186 10.1016/S1473-3099(15)00062-6 PMC4579482

[17] Stucki D (2012) Two new rapid SNP-typing methods for classifying *Mycobacterium tuberculosis* complex into the main phylogenetic lineages. PLoS ONE 7, e41253. 22911768 10.1371/journal.pone.0041253 PMC3401130

[18] The CRyPTIC Consortium and the 100,000 Genomes Project (2018) Prediction of susceptibility to first-line tuberculosis drugs by DNA sequencing. New England Journal of Medicine 379, 1403–1415. 30280646 10.1056/NEJMoa1800474 PMC6121966

[19] Nava-Aguilera E (2009) Risk factors associated with recent transmission of tuberculosis: systematic review and meta-analysis. The International Journal of Tuberculosis and Lung Disease 13, 17–26. 19105874

[20] Stein RA (2011) Super-spreaders in infectious diseases. International Journal of Infectious Diseases 15, e510–e513. 21737332 10.1016/j.ijid.2010.06.020 PMC7110524

[21] Mathema B (2017) Drivers of tuberculosis transmission. The Journal of Infectious Diseases 216, S644–S653. 29112745 10.1093/infdis/jix354 PMC5853844

[22] Cheng S (2013) Effect of diagnostic and treatment delay on the risk of tuberculosis transmission in Shenzhen, China: an observational cohort study, 1993–2010. PLoS ONE 8, e67516. 23826313 10.1371/journal.pone.0067516 PMC3694886

[23] MacIntyre CR (1995) High rate of transmission of tuberculosis in an office: impact of delayed diagnosis. Clinical Infectious Diseases 21, 1170–1174. 8589138 10.1093/clinids/21.5.1170

[24] Wlodarska M (2015) A microbiological revolution meets an ancient disease: improving the management of tuberculosis with genomics. Clinical Microbiology Reviews 28, 523–539. 25810419 10.1128/CMR.00124-14 PMC4402953

[25] Walker TM (2014) Assessment of *Mycobacterium tuberculosis* transmission in Oxfordshire, UK, 2007–12, with whole pathogen genome sequences: an observational study. The Lancet Respiratory Medicine 2, 285–292. 24717625 10.1016/S2213-2600(14)70027-X PMC4571080

