# Supplementary figures and images for: Whole genome sequencing for improved understanding of Mycobacterium tuberculosis transmission in a remote circumpolar region
Source: Epidemiol Infect. 2019 May 9;147:e188. doi: 10.1017/S0950268819000670 (PMC6518594; doi:10.1017/S0950268819000670)

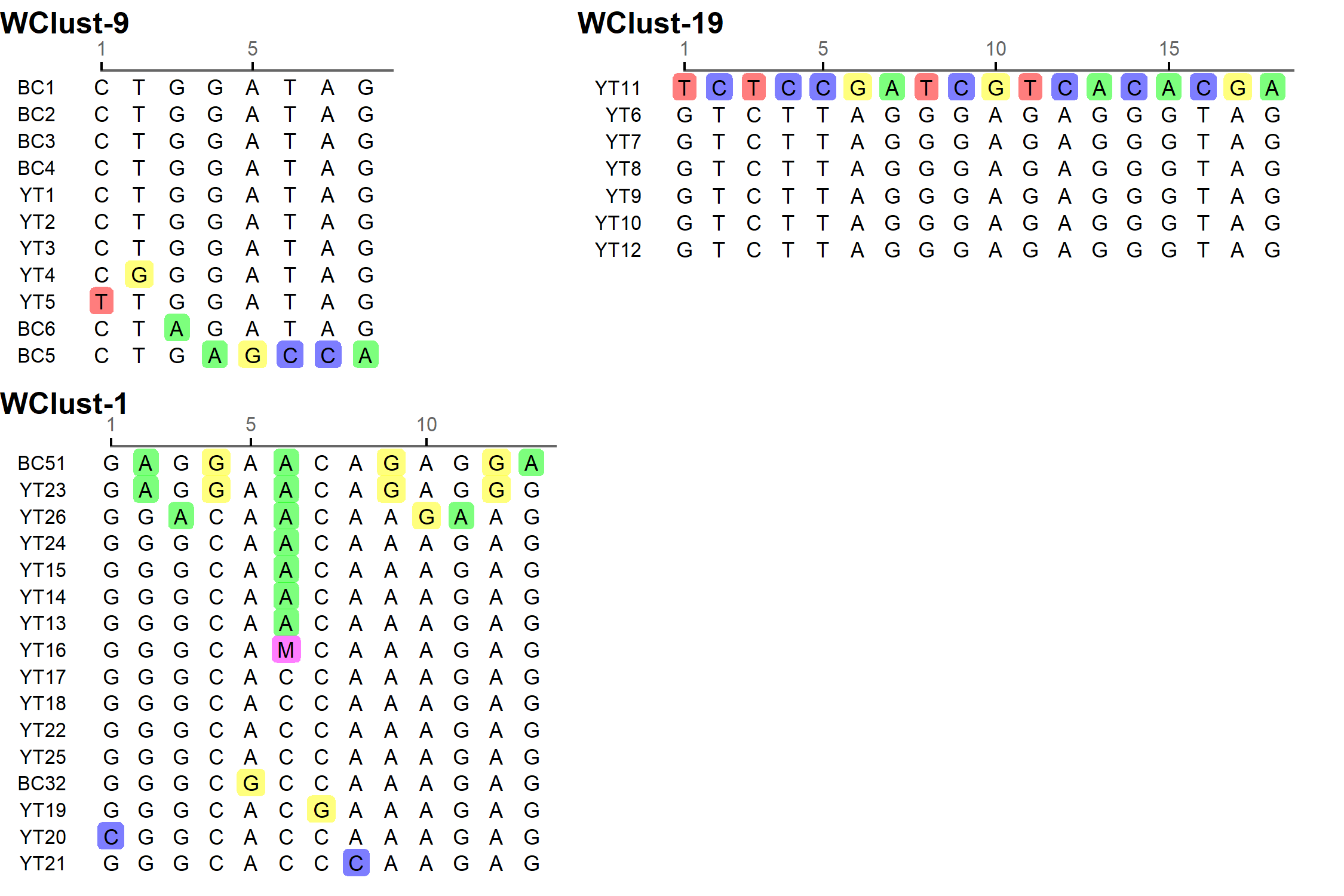

Supplement: Supplementary file 1 [file S0950268819000670sup001.png]
